# Effect of a Co-created Esports Interaction Program on Healthcare Professional Students’ Empathy Toward People With Early-Onset Dementia: A Pilot Study

**DOI:** 10.7759/cureus.89087

**Published:** 2025-07-30

**Authors:** Kohei Okuyama, Miho Hamayoshi, Megumi Abe, Atsuko Yasumoto

**Affiliations:** 1 Department of Physical Therapy, Bukkyo University, Kyoto, JPN; 2 Department of Nursing, Bukkyo University, Kyoto, JPN

**Keywords:** co-creation, early-onset dementia, empathy, esports, healthcare professional education, person-centered care

## Abstract

Background: Although empathy is crucial for the care of people with early-onset dementia (EOD), effective educational methods to cultivate it in healthcare professional students are not fully developed. This study evaluates a novel program using co-created esports to foster empathy.

Objective: This study aimed to investigate the potential effect of a co-created esports interaction program with people with EOD on the empathy of healthcare professional students.

Methods: In this single-group pre-post study, 35 healthcare professional students participated in a six-month program, conducted at Bukkyo University and Sanga Stadium by KYOCERA - e‑Sports Zone (Sky Field) in Kyoto, Japan. The program involved collaboratively planning and engaging in esports activities with people with EOD. Empathy was assessed before and after the program using the Jefferson Scale of Empathy for Health Profession Students (JSE-HPS). Pre- and post-program scores were compared using a paired t-test.

Results: The total JSE-HPS score significantly increased from 109.74 ± 12.72 to 117.69 ± 10.12 (t(34) = 4.03, p < 0.001). A sub-analysis revealed a significant improvement in the “Compassionate Care” subscale (p < 0.001), an indicator of emotional empathy. However, no significant changes were found in the cognitive empathy subscales, “Perspective-Taking” and “Walking in Patient’s Shoes.”

Conclusions: A participatory interaction program centered on a co-created, enjoyable activity such as esports may enhance emotional, but not cognitive, empathy in healthcare professional students toward people with EOD. Inclusive, activity-based educational models therefore hold promise for fostering compassionate care, while targeted strategies are still needed to strengthen cognitive empathy.

## Introduction

Dementia is a growing public health challenge worldwide [1]. Among its forms, early-onset dementia (EOD), which affects people under the age of 65, presents a distinct set of profound challenges [2-4]. Occurring typically during a person’s prime working and family life, EOD often leads to career interruptions, financial strain, and significant shifts in family dynamics [5], imposing a complex psychosocial burden on both the people affected and their families [6]. These unique complexities faced by people with EOD suggest that healthcare professional students may require a nuanced empathetic understanding, potentially different from that needed for older adults with dementia, to address their specific life challenges effectively.

High-quality, person-centered care for people with EOD necessitates deep empathy from healthcare professionals, encompassing both the cognitive ability to understand their experiences and the affective capacity to share their emotional state [7]; such empathy is consistently linked to improved patient satisfaction and therapeutic outcomes [8]. However, traditional dementia education for healthcare professional students often emphasizes didactic learning and may not sufficiently cultivate the empathetic skills needed for diverse populations like those with EOD. Meaningful, direct interaction with people with dementia is increasingly recognized as crucial for fostering genuine empathy and positive attitudes, moving beyond textbook knowledge to experiential understanding [9].

Existing research on interaction programs has predominantly focused on older adults with dementia, leaving a gap in understanding the impact of engagement with people with EOD on students’ empathy. Given that people with EOD are often younger and may retain a strong desire for active social participation, participatory interaction strategies that align with their interests could foster higher-quality engagement and empathetic development.

One such strategy could involve esports (electronic sports), which has emerged as a promising tool for promoting social interaction, cognitive engagement, and psychological well-being across different age groups, including older adults and people with cognitive impairments [10,11]. For a medical audience, it is important to distinguish esports from casual gaming; esports are organized, competitive video games that demand high levels of skill, strategic thinking, and, crucially, teamwork and communication [12]. This makes them a powerful medium for structured social interaction.

Esports is particularly well-suited for people with EOD for several reasons. First, from a generational perspective, many individuals with EOD grew up during the rise of home video games and possess a lifelong familiarity [13], which lowers psychological barriers to engagement. Second, it aligns with a strengths-based approach, allowing participants to utilize preserved cognitive abilities rather than focusing on deficits. Third, the team-based nature of many esports provides an opportunity to assume a meaningful and egalitarian social role as a "teammate," countering the social isolation and role loss often associated with an EOD diagnosis [14].

Therefore, this pilot study aimed to investigate the impact of an innovative interaction program, centrally featuring co-created esports activities, on the empathy levels of undergraduate healthcare professional students. We hypothesized that through the process of collaboratively planning and participating in esports (an activity aligned with the interests and strengths of people with EOD), students would enhance both their emotional and cognitive empathy. This study addresses a critical gap by evaluating a novel, age-appropriate, and strengths-based educational model. The findings are expected to contribute to the development of programs that can better prepare future healthcare professionals to provide empathetic and effective care for people with EOD, thereby promoting a more inclusive society.

## Materials and methods

Study design

The study employed a single-group pretest-posttest design without a control group. As a pilot study investigating the feasibility and potential efficacy of a novel intervention, this design was chosen to gather preliminary evidence before undertaking a larger randomized controlled trial. The study was conducted over a six-month period from July 2024 to December 2024. 

Introductory dementia‑supporter course and all survey administration were held at Bukkyo University (Nijo Campus, Kyoto, Japan), as well as two of the four co‑creation workshops. Meanwhile, two of the four co‑creation workshops and the culminating esports tournament were conducted at Sanga Stadium by KYOCERA - e‑Sports Zone (Sky Field), Kyoto, Japan.

Participants

Using a convenience sampling method, a total of 35 undergraduate students enrolled in healthcare professional programs participated in the study. Volunteers were recruited via posters and flyers distributed throughout the university. Eligibility criteria included current enrollment in a healthcare-related faculty and willingness to participate in all components of the intervention; no additional exclusion criteria were specified. Participants who withdrew before completing the baseline assessment were excluded from the study. An a priori power analysis (G*Power 3.1.9.6; effect size d = 0.5, α = 0.05, power = 0.80) indicated that 34 participants would be required to detect a moderate effect; 35 students volunteered and were enrolled, providing a small buffer for potential attrition.

Data collection

Data collection was conducted via self-administered web-based surveys. All surveys were administered in Japanese. To ensure anonymity, participants were assigned random ID numbers. They accessed the web-based survey via a QR code provided on the research information sheet and entered their assigned ID to link the pre- and post-program questionnaires. Responses to the web-based survey using Google Forms (Google LLC, Mountain View, California, United States) were requested at two time points: before participation in the interaction program and immediately after completing all sessions.

Evaluation measures

Participant characteristics (age, gender, academic department, and year of study) were recorded. Empathy was assessed with the authorized Japanese translation of the Jefferson Scale of Empathy (JSE)-Health Professions Students' version (JSE-HPS) [7], used with permission from Thomas Jefferson University.

The JSE comprises 20 items rated on a seven-point Likert scale (1 = strongly disagree, 7 = strongly agree), yielding total scores of 20-140, with higher scores indicating greater empathy. Ten items are positively worded and 10 are negatively worded, the latter being reverse-scored. The scale categorizes empathy into three factors: Perspective-Taking (cognitive understanding of another's viewpoint), Compassionate Care (other-oriented concern), and Walking in Patient's Shoes (experiential grasp of the recipient's internal world) [15]. Systematic reviews indicate that the various versions of the JSE are the most widely used instruments for assessing empathy in healthcare education and practice [16] and that the scale has been translated into more than 40 languages and applied in over 60 countries. Although a peer-reviewed psychometric study of the Japanese JSE-HPS has not yet been published, the closely related Japanese medical student version (JSE-S) has demonstrated satisfactory reliability (Cronbach's α ≥ 0.80) and a stable three-factor structure in a large cohort of medical students [17]; these findings were corroborated across an 11-year series of consecutive cohorts [18]. Cronbach's α for the Japanese JSE-HPS was calculated for the present sample as a quality check.

Description of the interaction program

The interaction program was conducted over a total of six months, with one main session held each month. The six-month duration was established to allow for the development of meaningful rapport and trust between students and participants with EOD, while the monthly frequency was chosen to ensure consistent engagement while respecting time commitments. The program consisted of the following three phases:

Phase 1: Introductory Session (Month 1)

The program began in the first month with a 90-minute Introductory Dementia-Supporter Course. Participants received core instruction on dementia pathophysiology, its psychosocial impact, and principles of person-centered care. A testimonial segment allowed individuals with EOD and their family members to share their day-to-day experiences and coping strategies, establishing the experiential framework for subsequent co-creation activities.

Phase 2: Monthly Co-Creation Workshops (Months 2-5)

**Figure 1:**
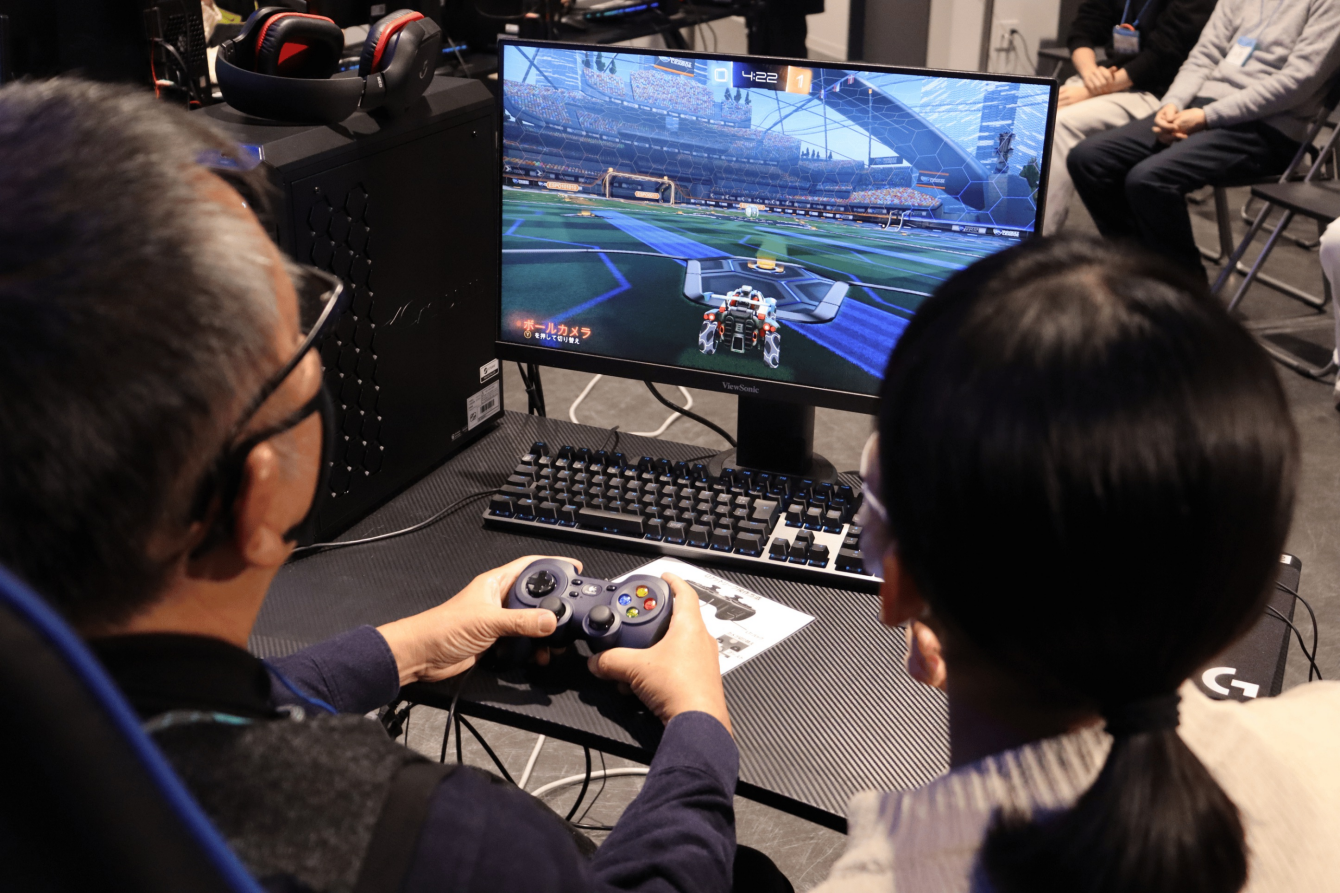
Co-creation workshop in session A participant with early-onset dementia and a healthcare student jointly playing Rocket League during the co-creation workshop.

From the second through the fifth month, four 90-minute co-creation workshops were held, one per month. At the first workshop, people with EOD voiced a clear desire to try an esports-based activity. In response, each subsequent monthly session followed a pragmatic cycle where students and participants with EOD collaborated in small groups for idea generation; hands-on trials of candidate games (including Rocket League (Psyonix LLC, San Diego, CA, USA), Puyo Puyo (SEGA Corporation, Tokyo, Japan), and Street Fighter 6 (Capcom Co. Ltd., Osaka, Japan)); and group reflection on accessibility, enjoyment, and perceived difficulty (Figure 1). Feedback from these discussions was used to co-creatively refine game rules, session length, and support strategies. This co-creative process, with esports as the central theme, was designed to foster collaborative problem-solving and shared decision-making.

Phase 3: Culminating Tournament (Month 6)

The program concluded in the sixth month with a 300-minute culminating social-exchange event: an esports tournament based on Rocket League. The tournament featured mixed teams (three versus three, ≥1 participant with EOD per team). Rocket League was selected because the EOD participants identified it as interesting and accessible, and because its dynamic, co-operative gameplay promotes engagement, teamwork, and real-time communication. The event served as a capstone opportunity for empathic interaction between students and the dementia community, reinforcing the program’s educational objectives while providing an inclusive and enjoyable social setting built upon months of collaborative esports planning and practice.

Statistical analysis

A paired t-test was conducted to compare the total JSE-HPS scores at two time points: before and after the interaction program. Additionally, as a sub-analysis, changes in JSE-HPS scores were analyzed for the three factors ("Perspective-Taking," "Compassionate Care," and "Walking in Patient's Shoes") to supplement the investigation of changes in empathy from each factor. To account for the effects of multiple comparisons, a Bonferroni correction was applied, setting the significance level at 0.0167. The assumption of normality for the paired t-test was assessed using the Shapiro-Wilk test on the difference scores, which indicated no significant deviation from normality (p > 0.05).

Furthermore, a one-way analysis of variance (ANOVA) was conducted to examine differences in empathy score changes across gender, department, and academic year. All analyses were conducted on an intention-to-treat (ITT) approach. This principle dictates that all 35 enrolled participants are included in the analysis according to the group to which they were originally assigned, regardless of their adherence to the program or completion of the post-test. This approach minimizes selection bias from participant attrition and provides a more realistic estimate of the program's effect in a real-world setting. Missing data were handled using multiple imputation methods.

Statistical analyses were performed using IBM SPSS Statistics for Windows, version 28.0 (IBM Corp., Armonk, NY, USA). The significance level for the primary paired t-test and the one-way ANOVA was set at p < 0.05.

Ethical considerations

Before the study, all participants received written and verbal explanations of the study’s purpose and procedures via an information sheet and provided written informed consent by signing a consent form. This consent also covered permission to capture and publish non-identifiable photographs taken during the sessions in which participants appeared. The protocol was approved by the Bukkyo University Human Research Ethics Review Committee (approval No. 2023-52-B), and the study was conducted in accordance with the Declaration of Helsinki, which states that participation is voluntary and that consent may be withdrawn at any time without penalty.

## Results

Participant characteristics and flow

A total of 35 undergraduate students were enrolled in the study (mean age 20.8 ± 1.1 years). The sample comprised 19 males (54.3%) and 16 females (45.7%). All 35 students completed the baseline assessment, whereas 33 (94.3%; 18 males, 15 females) completed the post-intervention assessment. In accordance with the ITT approach, all 35 enrolled participants were included in the final analysis; missing post-test data were handled by multiple imputation. Detailed demographic information is provided in Table 1.

**Table 1:** Participant characteristics (n = 35) Continuous variables are presented as mean ± SD; categorical variables as n (%); SD, standard deviation.

Characteristic	Category	Value (mean ± SD or n %)
Age		20.8 ± 1.1
Gender	Male	19 (54.3)
	Female	16 (45.7)
Academic year	Year 1	1 (2.9)
	Year 2	14 (40.0)
	Year 3	10 (28.6)
	Year 4	10 (28.6)
Department	Physical Therapy	21 (60.0)
	Nursing	9 (25.7)
	Occupational Therapy	5 (14.3)

Scale reliability

The Japanese JSE-HPS demonstrated good internal consistency at pretest (Cronbach’s α = 0.83, 95% CI 0.70-0.90; n = 35).

Changes in empathy scores

As detailed in Table 2, the total JSE-HPS score significantly improved from the pre-program mean of 109.74 ± 12.72 to the post-program mean of 117.69 ± 10.12 (t(34) = 4.03, p < 0.001, Cohen’s d = 0.68).

**Table 2:** Change in empathy score before and after the interaction program † p < 0.0167 after Bonferroni correction (subscale comparisons only); n = 35; JSE–HPS, Jefferson Scale of Empathy–Health Profession Students’ version; SD, standard deviation.

JSE–HPS score (points)	Pre-interaction program (Mean ± SD)	Post-interaction program (Mean ± SD)	t-value	p-value	Cohen’s d
Total	109.74 ± 12.72	117.69 ± 10.12	4.03	<0.001	0.68
Perspective-Taking	58.66 ± 6.95	61.06 ± 5.27	2.17	0.037	0.37
Compassionate Care	44.06 ± 7.55	48.63 ± 4.89	3.85	<0.001^†^	0.65
Walking in Patient’s Shoes	7.43 ± 3.16	8.00 ± 2.56	1.16	0.252	0.20

An analysis of the subscales revealed that “Compassionate Care” was the only factor to show a significant improvement (p < 0.001). No significant changes were observed for the “Perspective-Taking” (p = 0.037) or “Walking in Patient’s Shoes” (p = 0.252) subscales.

Subgroup analysis

The magnitude of change in total empathy scores did not differ significantly by gender, academic department, or academic year. The detailed results of the one-way ANOVA are presented in Table 3.

**Table 3:** Subgroup analysis of changes in total JSE-HPS scores All F tests were non‑significant (p > 0.05); df, degrees of freedom; partial η^2^, partial eta‑squared; JSE-HPS, Jefferson Scale of Empathy for Health Profession Students.

Factor	F	df_1_	df_2_	p	Partial η^2^
Gender	0.59	1	33	0.448	0.018
Academic department	0.03	2	32	0.968	0.002
Academic year	1.58	3	31	0.215	0.133

## Discussion

This pilot study offers preliminary evidence that a six‑month, co‑created esports program can enhance emotional empathy toward people with EOD in undergraduate healthcare students. The total JSE‑HPS score increased by 7.9 points (Cohen’s d = 0.68), a gain driven almost entirely by the Compassionate Care factor, whereas Perspective‑Taking and Walking in Patient’s Shoes did not reach the Bonferroni‑adjusted significance threshold. These findings partially confirm our a priori hypothesis: the intervention fostered affective empathy but did not yield measurable cognitive change within the study period.

The affect‑selective pattern can be explained by three distinctive features of esports. First, the activity created an egalitarian “teammate” relationship, in contrast to traditional direct-contact interventions (e.g., nursing‑home shadowing, doll therapy, or reminiscence visits) where students act as helpers and people with dementia as recipients of care [19,20]. By requiring real‑time cooperation in a competitive setting, esports neutralized role asymmetry and prompted participants to view one another as peers striving toward a shared goal. Second, the program was explicitly co‑created: individuals with EOD selected candidate titles, modified rules, and negotiated fair‑play conventions, thereby exercising agency that is rarely afforded in conventional placements. Agency is a recognized precursor of empathic concern because it frames the partner as a competent social actor rather than a passive beneficiary [21]. Third, a lifelong familiarity with digital games among many individuals with EOD likely lowered psychological barriers and accelerated rapport [13]. Together, these elements generated shared positive affect (joint fun and accomplishment), which can activate the affective, other‑oriented dimension of empathy more readily than didactic teaching alone [22].

By contrast, cognitive empathy may require structured reflection that was not embedded in the fast‑paced workshops. Perspective‑Taking often develops through deliberate metacognitive exercises [23]; without prompts to articulate what they had learned about the lived reality of EOD, students may have struggled to translate emotional resonance into cognitive insight. Future cohorts could append short debriefing circles, guided journals, or immersive virtual‑reality simulations immediately after gameplay to consolidate perspective‑taking skills [24,25]. Such add‑ons are inexpensive, impose minimal time burden, and can be delivered online, making them an attractive refinement for larger trials.

Esports also differs from conventional physical sports in ways that may widen access for people with functional limitations [26]. Although both formats entail teamwork and competition, esports relies primarily on cognitive‑strategic skills and fine motor control, not gross physical exertion. That distinction renders the activity inclusive for participants whose physical stamina or mobility might otherwise preclude equal play. At the same time, the linguistic and tactical communication required in games such as Rocket League mirrors the inter‑professional dialogue characteristic of contemporary healthcare [27], offering authentic practice in collaborative decision‑making.

The program’s design has several practical advantages for curriculum planners. A “4 + 1” structure (four 90‑minute co‑creation workshops held at four‑week intervals, followed by a three‑hour mixed‑team tournament) fits neatly into a 15‑week academic term. The hardware requirement is modest: entry‑level PCs or current‑generation consoles already present in many student lounges suffice, and the inherently networked nature of esports permits hybrid or fully remote delivery, extending reach to rural campuses at negligible marginal cost. Because each team must include at least one person with EOD, students from nursing, therapy, and medicine learn to negotiate roles in an authentically inter‑professional, strengths‑based environment. These characteristics suggest that the model is scalable, cost‑conscious, and consistent with modern pedagogical emphases on active, inclusive learning [28].

Important limitations temper these conclusions. The single‑group, pre-post design lacks a control arm, so history or maturation effects cannot be excluded; future work should employ randomized controlled designs. The sample was modest, self‑selected, and drawn from one university, limiting generalizability; cultural replication will be necessary. Self‑reported empathy is vulnerable to social‑desirability and Hawthorne effects, and no behavioral measures were obtained. Although the Japanese JSE‑HPS showed acceptable reliability, its full psychometric profile remains unpublished [16]. Moreover, the intervention is multifaceted (agency, competition, social bonding), and the study cannot disentangle which component drove the observed affective gain. Longer follow‑up (≥12 months) is required to establish durability, and cost‑effectiveness analyses would inform large‑scale adoption.

Despite these caveats, the present findings highlight the promise of co‑created, strengths‑based esports as an age‑appropriate vehicle for cultivating emotional empathy toward people with EOD. By reframing individuals with dementia as capable teammates rather than care recipients, the program fostered mutual respect and positive affect, conditions known to underlie compassionate care in clinical settings. If replicated and augmented with structured reflection to target cognitive empathy, such interventions could become a practical component of health‑care curricula aimed at preparing future professionals to deliver truly person‑centered dementia care.

## Conclusions

Despite its limitations as a pilot study, the present findings highlight the promise of a co-created, strengths-based esports program as an age-appropriate vehicle for cultivating emotional empathy toward people with EOD. By reframing individuals with dementia as capable teammates rather than passive care recipients, the program fostered mutual respect and positive affect, conditions known to underlie compassionate care. Future work employing randomized controlled designs, behavioral outcome measures, and longer follow-up is required to verify these preliminary effects and test their durability. Nevertheless, the study outlines a scalable, cost-conscious model; if replicated and augmented with structured reflection to target cognitive perspective-taking, such interventions could become a practical component of health-care curricula aimed at preparing professionals to deliver truly person-centered dementia care.
